# Supplementary material for: Serology Assays Used in SARS-CoV-2 Seroprevalence Surveys Worldwide: A Systematic Review and Meta-Analysis of Assay Features, Testing Algorithms, and Performance
Source: Vaccines (Basel). 2022 Nov 24;10(12):2000. doi: 10.3390/vaccines10122000 (PMC9783516; doi:10.3390/vaccines10122000)
Supplement: Supplementary file 1 [file vaccines-10-02000-s001.zip › vaccines-1967420-SM.pdf]

Supplementary file

Figure S1. PRISMA diagram

Table S1. The major categories of assay

Table S2. Reference panels of five third-party lab evaluations

Table S3. Testing characteristics of included seroprevalence studies

Figure S2. Pareto chart of serological assay use.

Figure S3. Difference in reported assay performance among independent evaluation, third party evaluation, and manufacturer evaluation among top 50 assays (ELISA, CLIA, and LFIA/IFA)

Figure S4. Pairwise Bland-Altman plots on the agreement of Sn. and Sp. between a source of assay evaluation against the manufacturer evaluation: Manufacturer vs. (a)-(b) Australia NRL, (c)-(d) US FDA, (e)-(f) Netherland CIDC, (g)-(h) FIND Diagnostics, (i)-(j) Australia Doherty, (k)-(l) independent evaluations

Figure S5. Pairwise Bland-Altman plots on the agreement of Sn. and Sp. between a source of lab evaluation against independent group evaluations: Independent vs. (a)-(b) Australia NRL, (c)-(d) US FDA, (e)-(f) Netherland CIDC, (g)-(h) FIND Diagnostics, (i)-(j) Australia Doherty.

Table S4. Categorizing the top 50 assays based on the median performance value given by third-party and independent evaluations

Table S5. Testing algorithms involving multiple serological assays

Figure S6. Bland-Altman plot on the agreement between serological testing results with multiple assays and with a single assay: 1) any single assay types (N = 167), 2) single assay: S-tests, 3) single assay: N-tests, 4) single assay: multiplexed tests, 5) single assay: RDTs, 6) single assay: ELISAs, 7) single assay: CLIAs, 8) single assay: LFIA/IFAs

Table S6. List of identified commercial serology assays from systematic review

Figure S1. PRISMA diagram

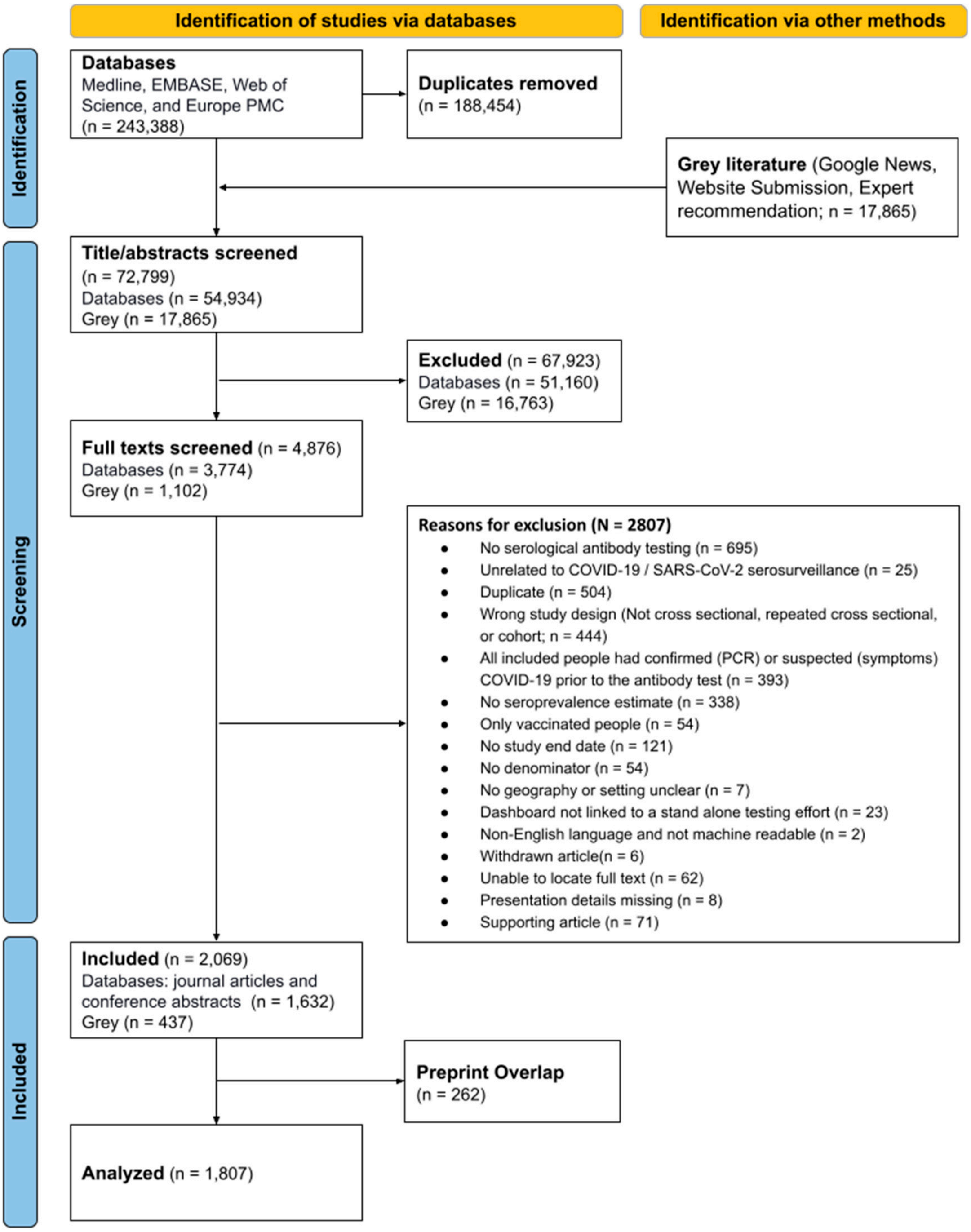

Table S1. The major categories of assay

Categories of assay	Description
Neutralization assays	Neutralization assays are lab-based tests that use live or pseudotyped viruses to determine if patient antibodies can prevent viral infection in vitro.
Lateral flow immunoassays (LFIAs)	LFIA technology is a type of rapid test, which is easily administered at the point of care by minimally trained personnel and at low cost, administered self with a rapid time-to-result turnaround.
Immunofluorescence assays (IFAs)	In IFAs, antibodies bind to the SARS-CoV-2 protein of interest. Fluorescent dyes are coupled to these immune complexes in order to visualize the protein of interest using microscopy or flow cytometry.
Enzyme-linked immunosorbent assays (ELISAs)	ELISA assay, also a lab-based test, is the most commonly used format of the serological test. Wells are coated with the viral antigen of interest. After the formation of immune complexes, an enzyme-linked secondary antibody followed by the enzyme's substrate is added, which results in chemical modification of the substrate in the presence of immune complexes, which is measured colorimetrically through spectroscopy. The relative light absorption at a particular wavelength is directly proportional to the number of labeled complexes present.
Chemiluminescence serology assays (CLIAs)	CLIA technology follows a similar concept to ELISA by taking advantage of high binding affinity between the viral antigen(s) and host antibodies but uses chemical probes that yield light responses that are directly proportional to the amount of labeled immune complexes present.
Other	Assays do not fall into the above five categories.

Table S2. Reference panels of five third-party lab evaluations

Third Party	Country	Time of Evaluation	Type of Assay	Isotype Detected	Overall Sample Size	Positive Sample	Negative Sample	Analysis
**FDA [1]**	USA	Since 2020 (Ongoing)	All types (including LFIAs, IFAs, ELISAs, CLIAs, and others, n = NA)	IgM, IgG, IgA, and total antibody	n >= 120	Confirmed with NAAT; disease severity unknown; time PSO unknown (*n* = 30)	Pre-pandemic negative serum and plasma (prior to 2020) without regard for clinical status (*n*=70); HIV+ (*n*=10)	Sensitivity calculated as the fraction of positive results among true positives, and specificity as the fraction of negative results among true negatives. Estimates were stratified by isotype (e.g., IgM, IgG, IgA, and total antibody) separately and combined where available.
**FIND Diagnostics [2,3]**	France, USA, Italy, Brazil, Spain, UK, Switzerla nd	March 2020 - March 2021	LFIAs (n=35) and ELISAs (n=16)	IgM, IgG, IgA, and total antibody	199 - 461	Confirmed with RT-PCR (*n* > 100); disease severity varies; time PSO (*n* = 10 for day 0-3, *n* = 20 for day 4-7, *n* = 30 for day 8-14, *n* = 20 for day 15-28, *n* = 20 for day 29+ by design but varied between site)	Pre-pandemic samples (prior to 2018; target *n* > 300)	Sensitivity calculated as the fraction of positive results among true positives, and specificity as the fraction of negative results among true negatives along with GLM fit with site and time PSO included as random effects. Estimates were stratified by time PSO, isotype (e.g., IgM, IgG, IgA, and total antibody) separately and combined where available.
**Netherland CIDC [4]**	Netherlan d	July 2020	LFIAs, IFAs, ELISAs, and CLIAs (n=47)	IgM, IgG, and total antibody	9 - 489	Confirmed with RT-PCR; disease severity varies; time PSO stratified by before or after 14 days (*n* varies between test and site)	Pre-pandemic samples (prior to Dec 2019); syndromic patients with potentially cross-reactive microorganisms during pandemic (*n* varies between test and site)	Sensitivity calculated as the fraction of positive results among true positives, and specificity as the fraction of negative results among true negatives (95% CI based on Wilson score). Sensitivity and specificity were stratified by disease severity, time PSO, isotype (e.g., IgM, IgG, IgA, and total antibody) separately and combined where available.

**Doherty Institute [5,6]**	Australia	April 2020 - April 2021	LFIA (n=27)	IgM, IgG, and total antibody	Varied in each phase of evaluation	Confirmed with RT-PCR; disease severity unknown; time PSO varies (*n* = 50-100, varies between the phase of evaluation)	Pre-pandemic samples (2018 and 2019) and non-COVID 19 infections (*n* = 92-100); cross-reactivity panel (*n* = 25; malaria, Influenza A, Influenza B, CMV IgM positive, acute parvovirus B19, EBV, mycoplasma, parainfluenza, C. psittaci, toxoplasma, rubella, and Hepatitis A and B)	Sensitivity calculated as the fraction of positive results among true positives, and specificity as the fraction of negative results among true negatives. Estimates were stratified by time PSO, isotype (e.g., IgM, IgG, and total antibody) separately and combined where available.
**NRL-WHO [7]**	Australia	Since December 2020 (Ongoing)	LFIAs, ELISAs, and CLIAs (n = 40)	IgG, IgM	NA	Confirmed with NAAT or clinical diagnosis; disease severity unknown; time PSO >14 days or >14 days post-NAAT (*n* unknown)	Pre-pandemic samples (prior to Nov 2019) (*n* unknown); cross-reactivity panel (*n* = 55; CMB IgM, EBV VCA IgM, Influenza A, Influenza B, Hepatitis A, Hepatitis B, Hepatitis C, HIV, malaria, mycoplasma, parainfluenza, parvovirus, C. psittaci, rubella, syphilis, toxoplasma)	Sensitivity calculated as the fraction of positive results among true positives, and specificity as the fraction of negative results among true negatives. Estimates were stratified by time PSO, isotype (e.g., IgM, IgG, and total antibody) separately and combined where available.

Table S3. Testing characteristics of included seroprevalence studies

Study Characteristics	Total (N = 1807)		Africa (N = 89)		Americas (N = 522)		Eastern Mediterranean (N = 98)		Europe (N = 846)		South-East Asia (N = 129)		Western Pacific (N = 123)	
	n	%	n	%	n	%	n	%	n	%	n	%	n	%
Sample Size (Median [IQR])	1240 (416-4558)		1769 (660-4258)		1096 (388-4445)		842 (388-2244)		1214 (386-4549)		1880 (728-10310)		1228 (512-6929)	
Sampling End Date[a]														
2020 Q1-Q2	668	37.0	12	13.5	187	35.8	32	32.7	361	42.7	11	8.5	65	52.8
2020 Q3-Q4	862	47.7	61	68.5	275	52.7	53	54.1	344	40.7	82	63.6	47	38.2
2021 Q1-Q2	248	13.7	16	18.0	55	10.5	12	12.2	128	15.1	27	20.9	10	8.1
2021 Q3-Q4	29	1.6	0	0.0	5	1.0	1	1.0	13	1.5	9	7.0	1	0.8
Specimen Type														
Serum	1016	56.2	34	38.2	257	49.2	66	67.3	489	57.8	82	63.6	88	71.5
Plasma	112	6.2	10	11.2	38	7.3	7	7.1	47	5.6	7	5.4	3	2.4
Whole blood	255	14.1	39	43.8	72	13.8	13	13.3	108	12.8	10	7.8	13	10.6
Dried blood	43	2.4	3	3.4	24	4.6	0	0.0	16	1.9	0	0.0	0	0.0
Multiple types	93	5.1	1	1.1	29	5.6	4	4.1	51	6.0	6	4.7	2	1.6
Not reported	288	15.9	2	2.2	102	19.5	8	8.2	135	16.0	24	18.6	17	13.8
No. of Test Used														
Single	1458	80.7	84	94.4	432	82.8	86	87.8	640	75.7	118	91.5	98	79.7
Multiple	349	19.3	5	5.6	90	17.2	12	12.2	206	24.3	11	8.5	25	20.3
Assay Types														
Commercial ELISA	655	36.2	26	29.2	174	33.3	30	30.6	357	42.2	39	30.2	29	23.6
Commercial LFIA	308	17.0	37	41.6	71	13.6	13	13.3	145	17.1	6	4.7	36	29.3
Commercial IFA	33	1.8	3	3.4	11	2.1	2	2.0	15	1.8	0	0.0	2	1.6
Commercial CLIA	879	48.6	12	13.5	258	49.4	47	48.0	428	50.6	63	48.8	71	57.7
Commercial Other[b]	14	0.8	0	0.0	5	1.0	0	0.0	8	0.9	0	0.0	1	0.8
Self-developed assay	167	9.2	5	5.6	31	5.9	8	8.2	95	11.2	2	1.6	26	21.1
Unknown	287	15.9	3	3.4	100	19.2	9	9.2	128	15.1	32	24.8	15	12.2
Test-adjusted estimation[c]														
Not Adjusted	1559	86.3	68	76.4	451	86.4	86	87.8	733	86.6	108	83.7	113	91.9
Adjusted	248	13.7	21	23.6	71	13.6	12	12.2	113	13.4	21	16.3	10	8.1

Abbreviations: ELISA: enzyme-linked immunosorbent assay; LFIA: lateral flow serology assay; IFA: immunofluorescence assays; CLIA: chemiluminescent serology assay; CMIA: chemiluminescent microparticle serology assay; RDT: rapid diagnostic tests.

Notes: [a] Q1-Q2 was between Jan. 1st and Jun. 30th of the given year; Q3-Q4 was between Jul. 1st and Dec. 31st of the given year.

[b] Other commercial serology assays include Luminex, and other test types were not specified by manufacturers.

[c] Bayesian methods were adopted to adjust for misclassification due to test Sn. / Sp. for more accurate prevalence estimations

Figure S2. Pareto chart of serological assay use.

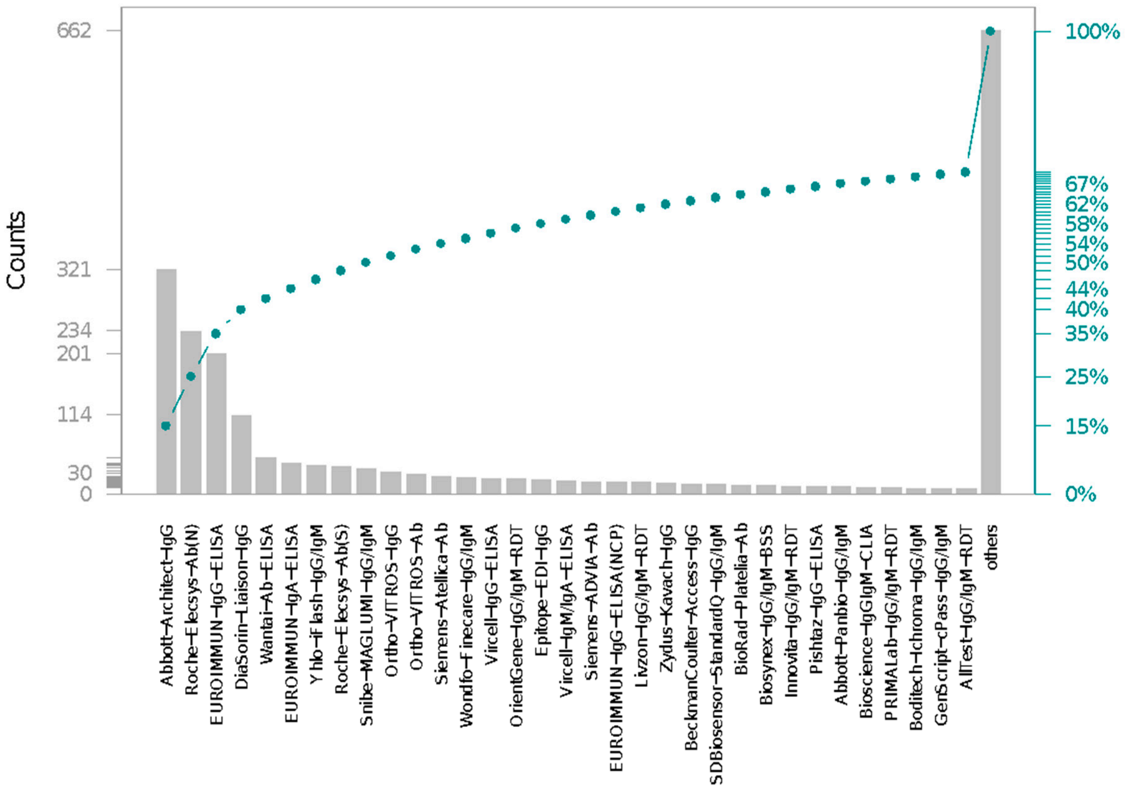

Note: Plot shows the frequency of serological assay use in 1807 serosurveys. Immunoassays are sorted by frequency of use. The top 25 immunoassays are shown individually with the remainder being grouped as "others".

Figure S3. Difference in reported assay performance among independent evaluation, third party evaluation, and manufacturer evaluation among top 50 assays (ELISA, CLIA, and LFIA/IFA)

(a) Difference in reported assay performance among independent evaluation, third party evaluation, and manufacturer evaluation among top 50 assays (ELISA)

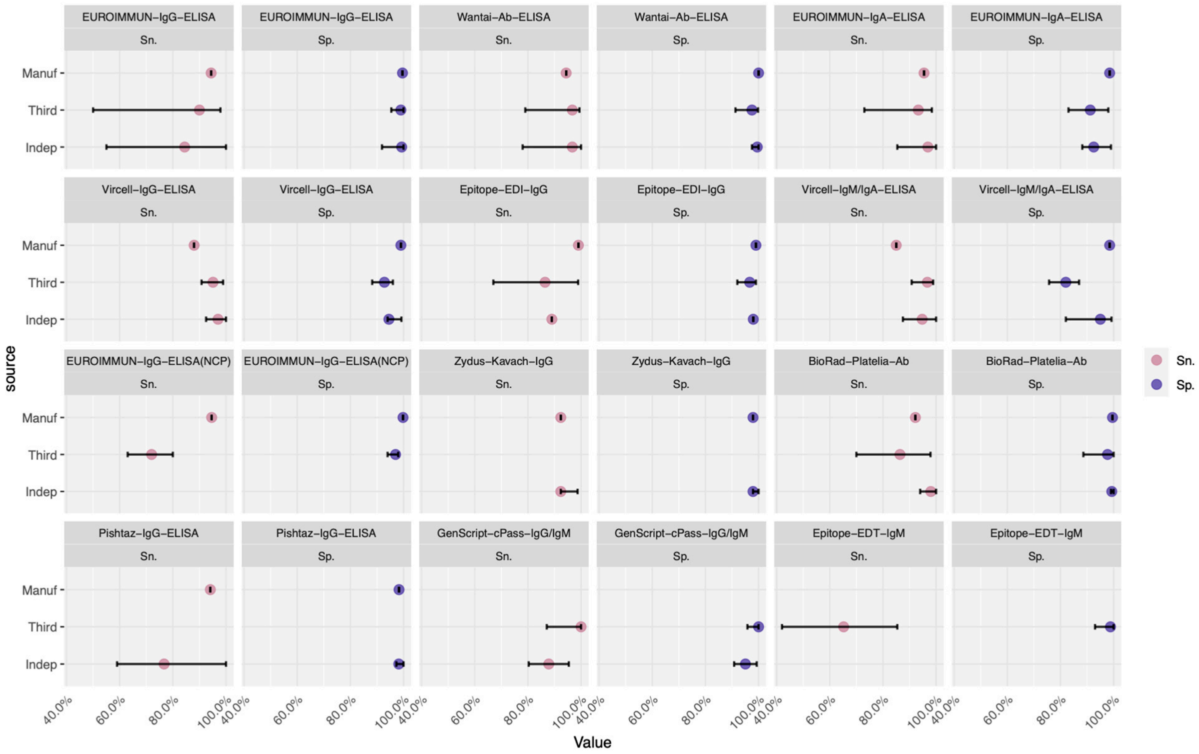

Note. The figure shows the side-by-side comparison of assay performance for the top 25 assays. Performance evaluations came from three sources: manufacturer reports, third-party labs and independent groups. Intervals were constructed by computing the minimum/maximum performance values from the given source.

(b) Difference in reported assay performance among independent evaluation, third party evaluation, and manufacturer evaluation among top 50 assays (CLIA)

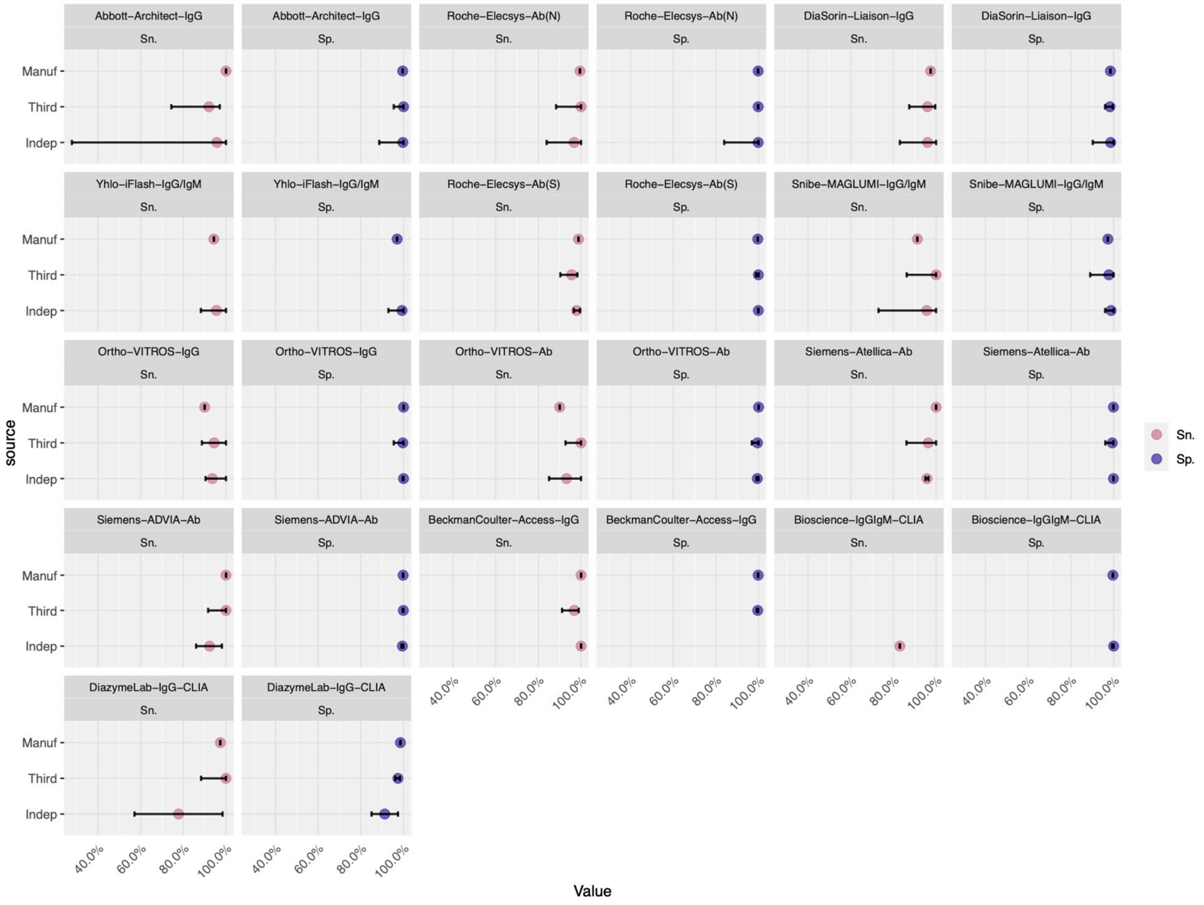

(c) Difference in reported assay performance among independent evaluation, third party evaluation, and manufacturer evaluation among top 50 assays (LFIA/IFA)

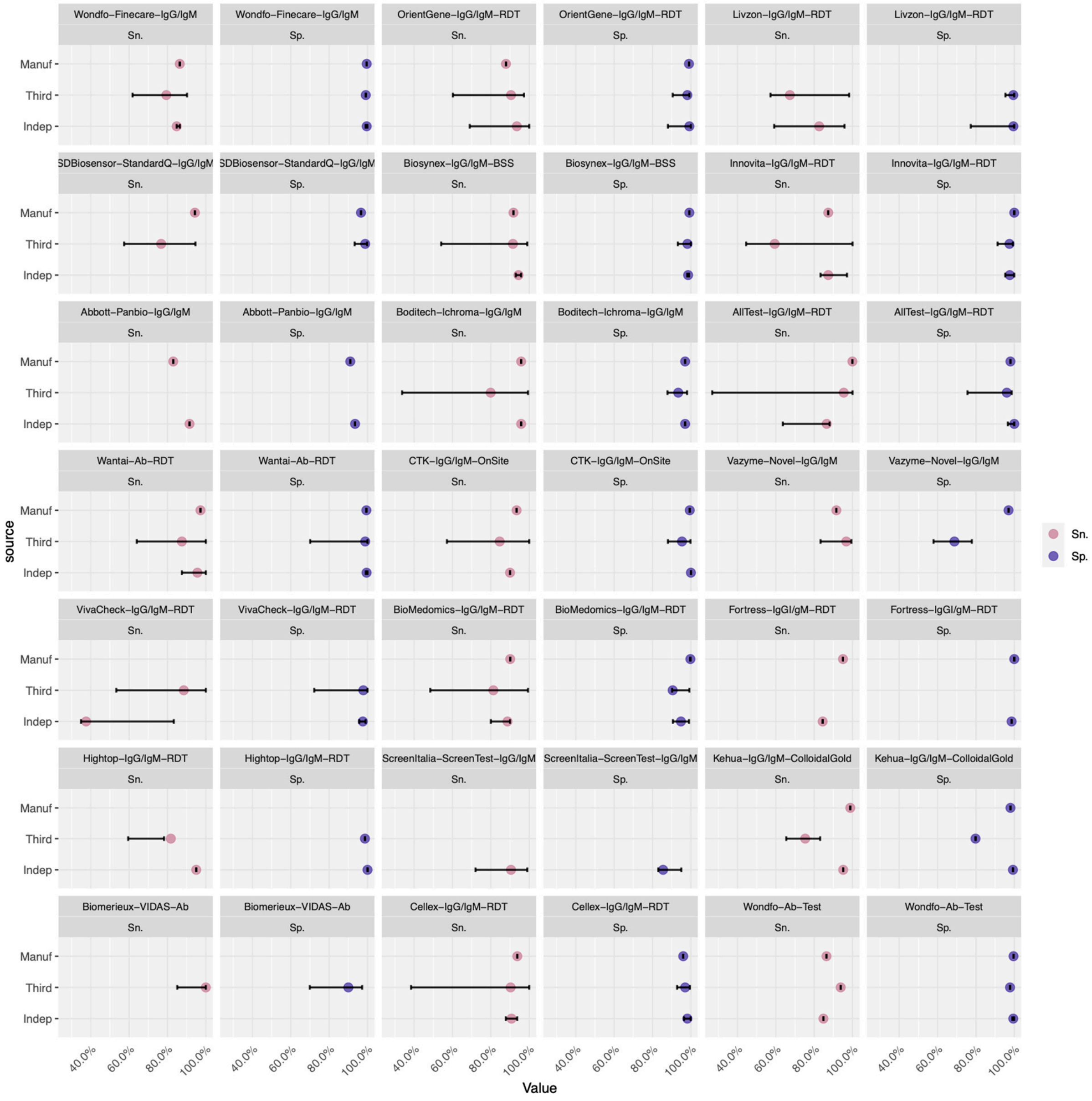

Figure S4. Pairwise Bland-Altman plots on the agreement of Sn. and Sp. between a source of assay evaluation against the manufacturer evaluation: Manufacturer vs. (a)-(b) Australia NRL, (c)-(d) US FDA, (e)-(f) Netherland CIDC, (g)-(h) FIND Diagnostics, (i)-(j) Australia Doherty, (k)-(l) independent evaluations

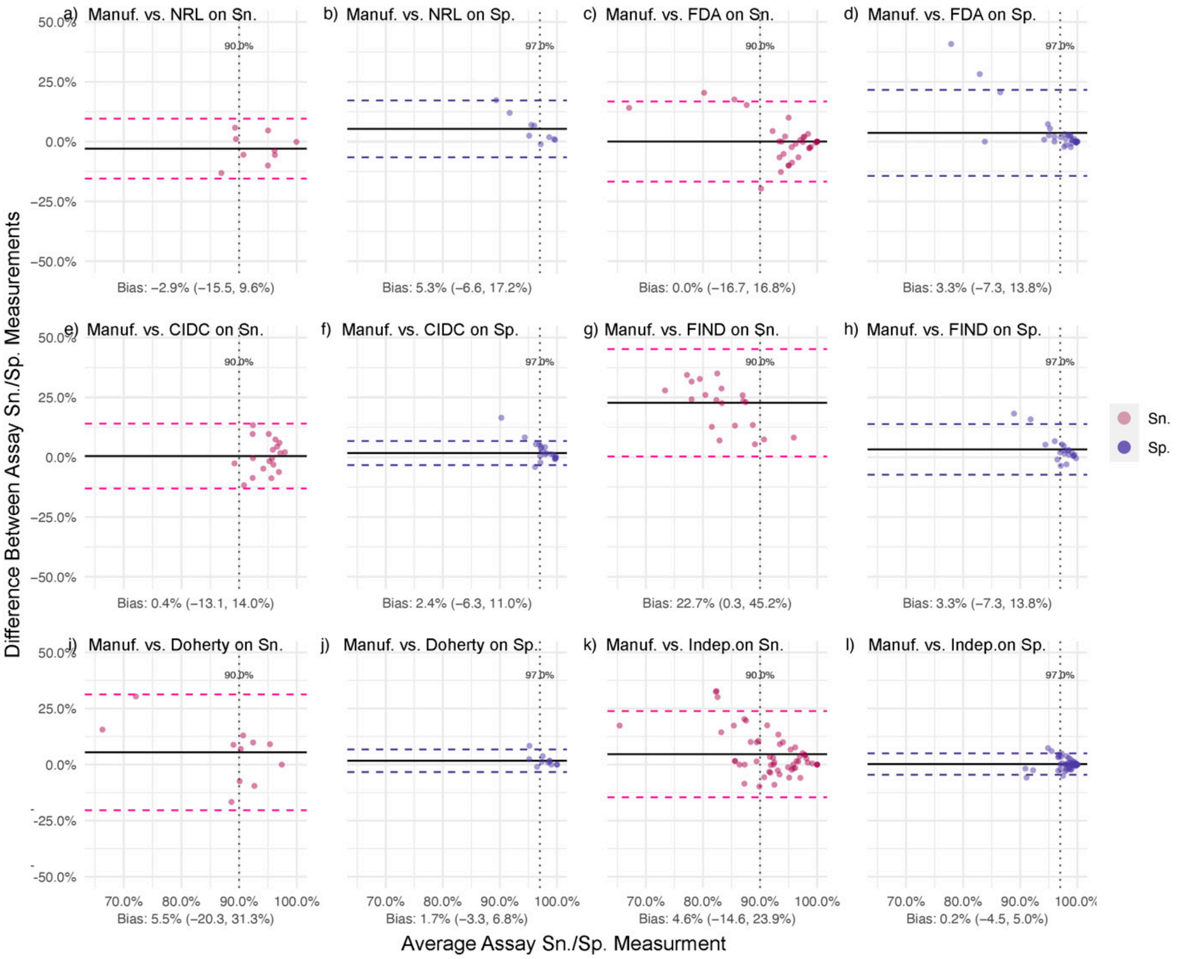

Note. Assays missing corresponding data were not involved in the analysis. The bias level (with its 95% confidence interval) was the average difference given by performance value from manufacturers - performance value from the other evaluation source.

Figure S5. Pairwise Bland-Altman plots on the agreement of Sn. and Sp. between a source of lab evaluation against independent group evaluations: Independent vs. (a)-(b) Australia NRL, (c)-(d) US FDA, (e)-(f) Netherland CIDC, (g)-(h) FIND Diagnostics, (i)-(j) Australia Doherty.

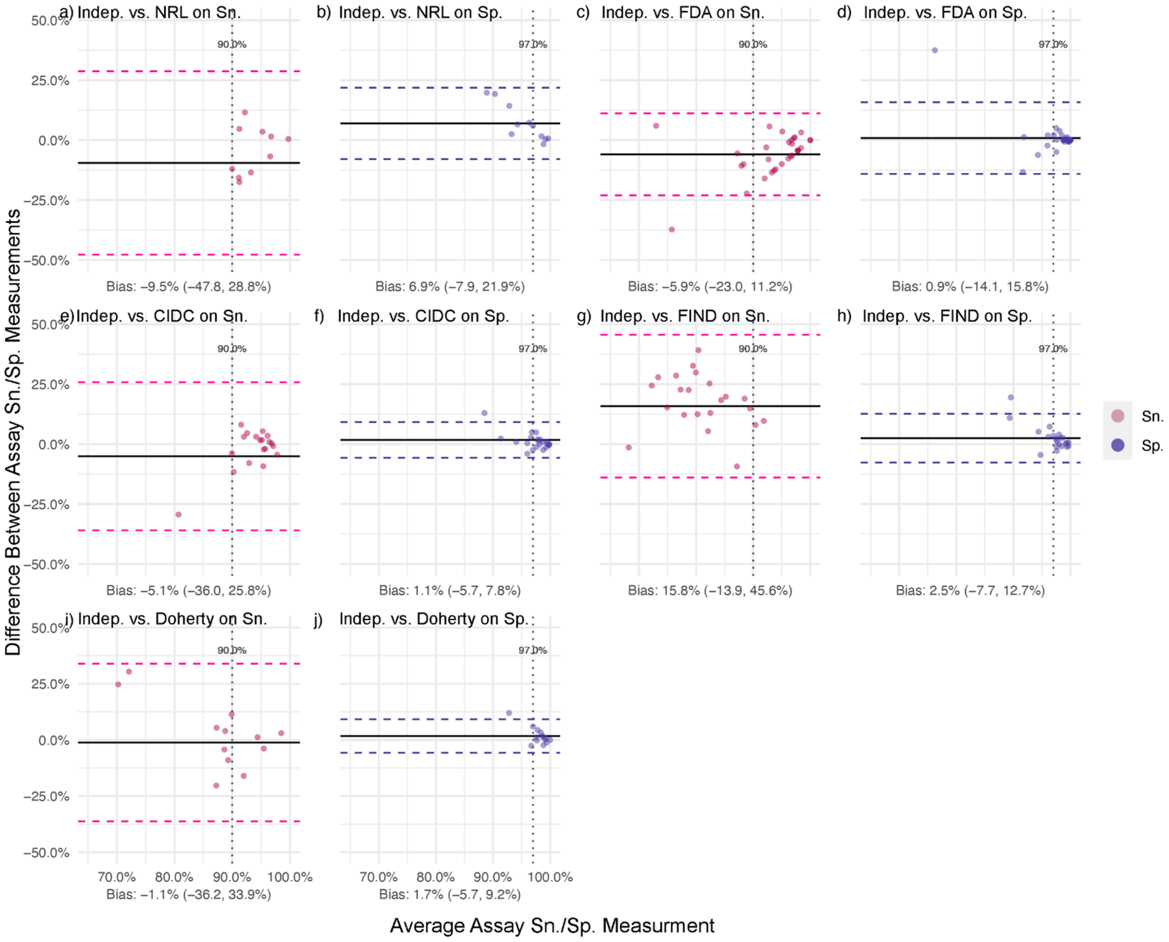

Note. Assays missing corresponding data were not involved in the analysis. The bias level (with its 95% confidence interval) was the average difference given by performance value from manufacturers - performance value from the other evaluation source

Table S4. Categorizing the top 50 assays based on the median performance value given by third-party and independent evaluations

Group	A (Sn: 95.0-99.9%, Sp: 97.0-99.9%; n = 10)	B (Sn: 90.0-95.0%, Sp: 90.0-97.0%; n = 8)	C (Sn: 80.0-90.0%, Sp: 87.0-90.0%; n = 18)	D (Sn: <80.0%, Sp: <87.0%; n = 18)
**Serological assays (Abbv.)**	Abbott-Architect-IgG, Roche-Elecsys-Ab(N), Yhlo-iFlash-IgG/IgM, GenScript-cPass-IgG/IgM, Roche-Elecsys-Ab(S), Wantai-Ab-ELISA, Siemens-Atellica-Ab, Ortho-VITROS-Ab, Siemens-ADVIA-Ab, BeckmanCoulter-Access-IgG	DiaSorin-Liaison-IgG, BioRad-Platelia-Ab, Snibe-MAGLUMI-IgG/IgM, PRIMALab-IgG/IgM-RDT, Ortho-VITROS-IgG, OrientGene-IgG/IgM-RDT, Biosynex-IgG/IgM-BSS, Wantai-Ab-RDT	EUROIMMUN-IgG-ELISA, Boditech-Ichroma-IgG/IgM, Bioscience-IgGIgM-CLIA, EUROIMMUN-IgA-ELISA, Cellex-IgG/IgM-RDT, Vircell-IgG-ELISA, Vircell-IgM/IgA-ELISA, Abbott-Panbio-IgG/IgM, Epitope-EDI-IgG, BioMedomics-IgG/IgM-RDT, Wondfo-Finecare-IgG/IgM, DiazymeLab-IgG-CLIA, CTK-IgG/IgM-OnSite, Biomerieux-VIDAS-Ab, Wondfo-Ab-Test, Fortress-IgGI/gM-RDT, Kehua-IgG/IgM-ColloidalGold, Hightop-IgG/IgM-RDT, Zydus-Kavach-IgG	AllTest-IgG/IgM-RDT, Innovita-IgG/IgM-RDT, Pishtaz-IgG-ELISA, VivaCheck-IgG/IgM-RDT, Livzon-IgG/IgM-RDT, EUROIMMUN-IgG-ELISA(NCP), Vazyme-Novel-IgG/IgM, ScreenItalia-ScreenTest-IgG/IgM, Epitope-EDT-IgM, SDBiosensor-StandardQ-IgG/IgM

Table S5. Testing algorithms involving multiple serological assays

Multiple Test Combination	Studies (N = 349)	
	n	%
No. of tests used		
Two tests	281	80.5
Three or more	68	19.5
Test types combined		
Commercial & Commercial	28	8.0
Commercial & Self-developed	67	19.2
Self-developed & Self-developed	254	72.8
Assay modalities combined		
RDT with RDT	20	5.7
RDT with Non-RDT (*)	82	23.5
Non-RDT with Non-RDT	267	76.5
Antibody targets combined		
S-Test with S-Test	121	34.7
S-Test with N-Test	152	43.6
Other/ Unknown	76	21.8
Combined Sn. and Sp.		
Reported	33	9.5
Not reported	283	81.1
Testing Algorithm		
Participants considered seropositive if at least one test is positive (Parallel OR)	101	28.9
Participants considered seropositive if all tests are positive (Parallel AND)	47	13.5
One test on all participants, with a second test used to confirm positives; participants considered seropositive if both tests were positive (Series AND)	111	31.8
Different singular tests (non-overlapping testing) used on different participants	30	8.6
Unclear	60	17.2

*Note: * Non-RDT tests included ELISA, CLIA (including CGIA and CGIA), and neutralization assays*

Figure S6. Bland-Altman plot on the agreement between serological testing results with multiple assays and with a single assay: 1) any single assay types (N = 167), 2) single assay: S-tests, 3) single assay: N-tests, 4) single assay: multiplexed tests, 5) single assay: RDTs, 6) single assay: ELISAs, 7) single assay: CLIAs, 8) single assay: LFIA/IFAs

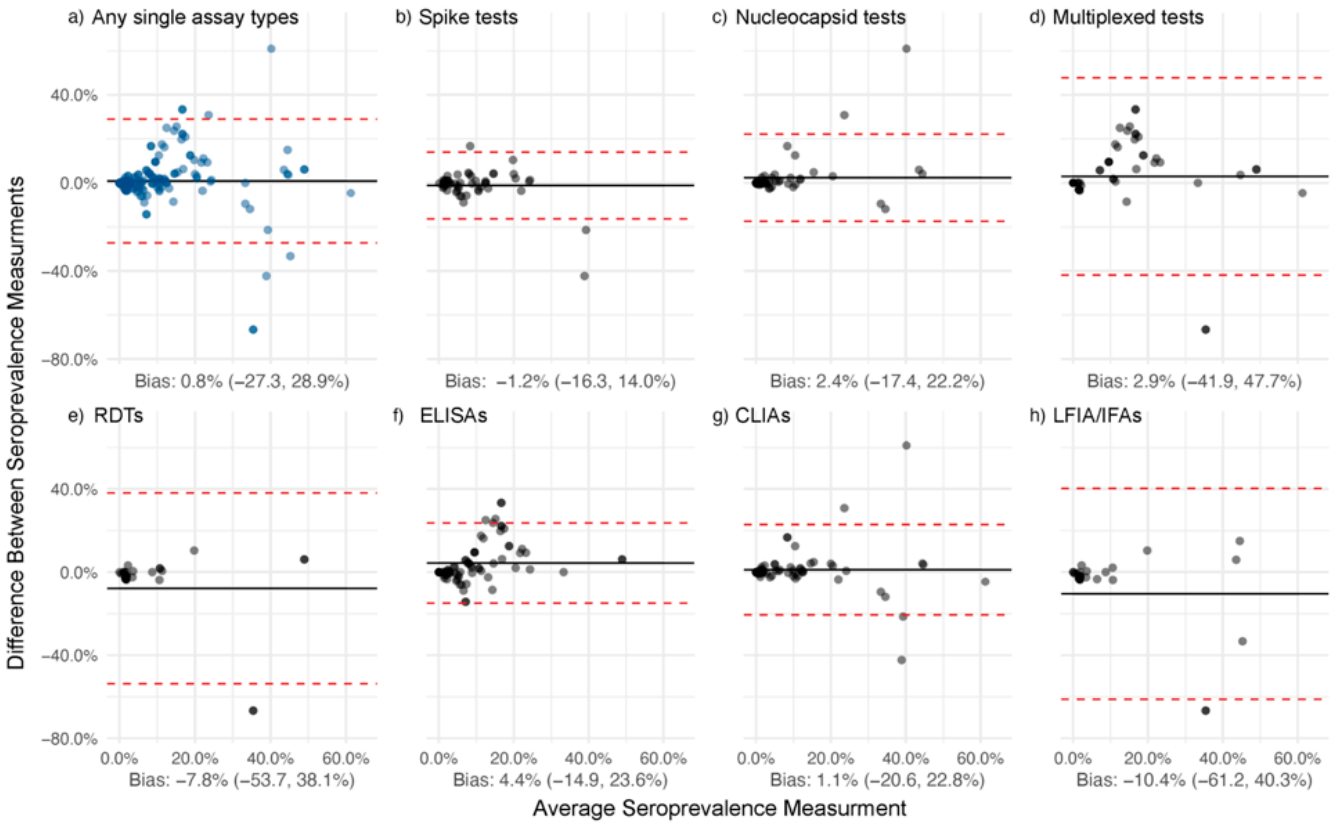

Note. Assays missing corresponding data were not involved in the analysis. The bias level (with 95% confidence interval) was the average difference given by seroprevalence estimated by the multiple testing - seroprevalence estimated by single testing.

Table S6. List of identified commercial serology assays from systematic review

No.	Manufacturer	Test Name	Country	Type	Isotype	Antibody Target	Sn. (Manuf., %)	Sp. (Manuf., %)	Sn.[1] (Third., %)	Sp. (Third., %)	Sn.[2] (Indep., %)	Sp. (Indep., %)
**1**	AAZ LMB	COVID-PRESTO	France	LFIA	IgG,IgM	Nucleocapsid	100.0	100.0			100.0 (100.0, 100.0)	100.0 (100.0, 100.0)
**2**	Abbexa	Abbexa COVID-19 IgG/IgM Rapid Test Kit	UK	LFIA	IgG,IgM	Spike	98.5	97.9	96.7 (83.3, 99.4)	96.7 (83.3, 99.4)	-	-
**3**	Abbott Laboratories	Abbott Architect SARS-CoV-2 IgG	USA	CLIA	IgG	Nucleocapsid	100.0	99.6	92 (74.4, 97.1)	92 (74.4, 97.1)	95.7 (27.8, 100.0)	99.7 (88.6, 100.0)
**4**	Abbott Laboratories	AdviseDX SARS-CoV-2 anti-IgG II assay	USA	CLIA	IgG	Spike	-	-	-	-	98.1 (98.1, 98.1)	99.6 (99.6, 99.6)
**5**	Abbott Laboratories	SARS-CoV-2 IgG II Quant	USA	CLIA	IgG	Spike	-	-	-	-	-	-
**6**	Abbott Laboratories	Panbio COVID-19 IgG/IgM rapid test device	USA	LFIA	IgG,IgM	Nucleocapsid	83.0	91.0	-	-	91.5 (91.5, 91.5)	93.5 (93.5, 93.5)
**7**	Abbott Laboratories	Architect Anti-SARS-CoV-2 IgM	USA	CLIA	others	Spike	-	-	-	-	-	-
**8**	Abnova	COVID-19 Human IgM IgG Assay Kit	China	ELISA	IgG,IgM	NA	94.4	98.5	-	-	-	-

[1,2] *The intervals represent the full ranges of Sn. and Sp. obtained by pooling all performance data from the given evaluation source (i.e., third-party labs, and independent groups). The point estimate is median of the performance value.*

**9**	Academia Sinica	Academia Sinica ELISA assay	China	ELISA	IgG,IgM	Spike	-	-	-	-	-	-
**10**	ACRO Biotech Inc	Acro Biotech COVID-19 IgM/IgG Rapid POC test	USA	LFIA	IgG,IgM	NA	-	-	91.9 (78.7, 97.2)	91.9 (78.7, 97.2)	88.0 (37.0, 96.0)	94.0 (79.2, 99.0)
**11**	Alfa Scientific Designs	Instant-view IgG/IgM Antibody COVID- 19 test	USA	LFIA	IgG,IgM	NA	97.8	94.6	100.0 (88.7, 100.0)	100.0 (88.7, 100.0)	-	-
**12**	AMEDA Labordiagnostik GmbH	AMP Rapid Test SARS-CoV-2 IgG/IgM	Austria	LFIA	IgG,IgM	NA	91.8	96.4	-	-	92.0 (92.0, 92.0)	99.4 (99.4, 99.4)
**13**	AnshLabs	SARS-CoV-2 IgG ELISA	USA	ELISA	IgG	Multiplex targets	-	-	-	-	-	-
**14**	Artron Laboratories Inc.	One Step Novel Coronavirus (COVID-19) IgM/IgG Antibody Test	Canada	LFIA	IgG,IgM	NA	93.4	97.7	96.2 (87.8, 98.2)	96.2 (87.8, 98.2)	83.3 (83.3, 83.3)	100.0 (100.0, 100.0)
**15**	Assure Tech. (Hangzhou) Co., Ltd	COVID-19 IgG/IgM Rapid Test Device	China	LFIA	IgG,IgM	Multiplex targets	-	-	99.5 (50.9, 100.0)	99.5 (50.9, 100.0)	100.0 (100.0, 100.0)	98.8 (98.8, 98.8)
**16**	Aurora Biomed Inc	COVID-19 IgG/IgM Rapid Test	Canada	LFIA	IgG,IgM	NA	-	-	76.7 (59.1, 88.2)	76.7 (59.1, 88.2)	-	-
**17**	Baiya Phytopharm	Baiya Rapid COVID-19 IgG/IgM Test Kit	Thailand	LFIA	IgG,IgM	Multiplex targets	-	-	-	-	94.1 (88.7, 94.1)	98.0 (90.63, 98.0)
**18**	Beckman Coulter	Access SARS-CoV-2 IgG Antibody Test	USA	CLIA	IgG	Spike	100.0	99.8	96.8 (91.1, 98.9)	96.8 (91.1, 98.9)	100.0 (100.0, 100.0)	-

**19**	Beijing Hotgen Biotech Co. Ltd, China	COVID-19 IgM/IgG Total Antibody Rapid Test	China	NA	IgG,IgM	NA	-	-	-	-	-	-
**20**	Beijing Lepu Medical Technology	Leccurate SARS-CoV-2 Antibody Rapid Test Kit (Colloidal Gold Immunochromatography)	China	LFIA	IgG,IgM	Nucleocapsid	98.8	97.6	-	-	66.0 (66.0, 66.0)	97.0 (97.0, 97.0)
**21**	Beijing Wantai Biological	Wantai SARS-CoV-2 IgG ELISA	China	ELISA	IgG	NA	-	-	100.0 (100.0, 97.64)	100.0 (100.0, 97.64)	82.5 (82.5, 82.5)	98.0 (98.0, 98.0)
**22**	Beijing Wantai Biological	Wantai Rapid Test for Total Antibody to SARS-CoV-2	China	LFIA	IgG,IgM,IgA	Spike	97.2	99.4			95.6 (87.5, 100.0)	99.5 (99.1, 100.0)
**23**	Beijing Wantai Biological	Wantai SARS-CoV-2 Total Ab ELISA	China	ELISA	IgG,IgM,IgA	Spike	94.4	100.0	96.7 (79.0, 99.4)	96.7 (79.0, 99.4)	96.7 (78.0, 100.0)	99.5 (97.5, 100.0)
**24**	Beijing Wantai Biological	Wantai SARS-CoV-2 IgM ELISA	China	ELISA	others	Spike	-	-	86.2 (69.0, 96.3)	86.2 (69.0, 96.3)	-	-
**25**	BGI Genomics	SARS-CoV-2 Virus IgG Antibody Detection Kit (ELISA)	China	ELISA	IgG	NA	98.7	98.4	-	-	-	-
**26**	Bio-rad	Platelia SARS-CoV-2 Total Ab assay	USA	ELISA	IgG,IgM,IgA	Nucleocapsid	92.2	99.6	86.4 (70.0, 97.9)	86.4 (70.0, 97.9)	98.0 (94.0, 100.0)	99.3 (99.0, 100.0)
**27**	Biohit Health Care Ltd	BIOHIT SARS-CoV-2 antibody test	UK	LFIA	IgG,IgM	NA	-	-	96.4 (83.3, 99.4)	96.4 (83.3, 99.4)	97.5 (97.5, 97.5)	100.0 (100.0, 100.0)

**28**	Biolidics Ltd	2019 nCoV IgM/IgG detection kit	Singapore	CLIA	IgG,IgM	Spike	-	-	96.7 (83.3, 99.4)	96.7 (83.3, 99.4)	-	-
**29**	BioM√©rieux	VIDAS SARS-COV-2 IgG test	France	other	IgG	Spike	-	-	100.0 (88.3, 100.0)	100.0 (88.3, 100.0)	-	-
**30**	BioMedomics, Inc.	COVID-19 IgM-IgG Dual Antibody Rapid Test	USA	LFIA	IgG,IgM	Spike	90.1	99.7	81.3 (48.4, 99.4)	81.3 (48.4, 99.4)	88.7 (80.0, 90.0)	94.8 (90.6, 99.0)
**31**	Biomerieux	VIDAS Multiparametric immunoassay system for medium throughput	France	FIA	IgG,IgM,IgA	Nucleocapsid	-	-	100.0 (85.1, 100.0)	100.0 (85.1, 100.0)	-	-
**32**	Bioscience Diagnostics	Magnetic Chemiluminescence Enzyme Immunoassay Kit	China	CLIA	IgG,IgM	Multiplex targets	-	99.7	-	-	83.0 (83.0, 83.0)	100.0 (99.3, 100.0)
**33**	Biosense Technologies, India	Coviscreen kittotal antibody (IgM+IgA+IgG)	India	NA	IgG,IgM,IgA	NA	100.0	99.0	-	-	-	-
**34**	Biosynex	Biosynex COVID-19 BSS assay	France	LFIA	IgG,IgM	Spike	91.8	99.2	91.5 (54.1, 99.0)	91.5 (54.1, 99.0)	94.4 (93.0, 95.8)	98.5 (98.1, 99.0)
**35**	BioSys Laboratories	The BioSys Plus COVID-19 IgM/IgG Rapid Test	Republic of Korea	LFIA	IgG,IgM	NA	93.5	100.0	-	-	-	-
**36**	Biozek	Biozek Medical COVID-19 Rapid Test	Netherlands	LFIA	IgG,IgM	NA	-	-	95.4 (90.3, 97.9)	95.4 (90.3, 97.9)	66.0 (66.0, 66.0)	97.0 (97.0, 97.0)

**37**	Boditech Med Inc.	AFIAS COVID-19 Ab assay	Republic of Korea	FIA	IgG,IgM	Multiplex targets	99.1	94.1	85.7 (48.7, 97.2)	85.7 (48.7, 97.2)	-	-
**38**	Boditech Med Inc.	Ichroma COVID-19 Ab Test	Republic of Korea	FIA	IgG,IgM	NA	95.8	97.0	79.9 (33.7, 99.4)	79.9 (33.7, 99.4)	95.8 (95.8, 95.8)	97.0 (97.0, 97.0)
**39**	Calbiotech Inc.	ErbaLisa COVID-19 IgG ELISA Kit	USA	ELIS A	IgG	NA	98.3	99.0	-	-	-	-
**40**	Cambridge Network	COVID-19 IgG and IgM Rapid Test	NA	LFIA	IgG,IgM	NA	-	-	-	-	100.0 (100.0, 100.0)	100.0 (100.0, 100.0)
**41**	Celer Technologies Inc.	One Step COVID-19 Test	Brazil	LFIA	IgG,IgM	NA	86.4	97.6	-	-	86.4 (86.4, 86.4)	99.6 (99.6, 99.6)
**42**	Cellex Inc.	COVID-19 IgG/IgM Rapid Test Cassette	USA	LFIA	IgG,IgM	Nucleocapsid	93.8	96.0	90.3 (38.4, 100.0)	90.3 (38.4, 100.0)	90.8 (87.8, 93.8)	98.2 (96.4, 99.9)
**43**	ChemBio Diagnostics Inc.	DPP COVID-19 IgM/IgG System	USA	NA	IgG,IgM	Nucleocapsid	-	-	82.1 (64.4, 92.1)	82.1 (64.4, 92.1)	-	-
**44**	CHiL	COVID-19 IgG/ IgM Rapid Test Cassette	Turkey	LFIA	IgG,IgM	Multiplex targets	-	-	-	-	-	-
**45**	CLUNGENE	CLUNGENE SARS-COV-2 VIRUS (COVID-19) IgG/IgM Rapid Test Cassette lateral flow immunoassay	Germany	LFIA	IgG,IgM	NA	-	-	-	-	-	-
**46**	CONICET	COVIDAR IgM and IgG ELISA	Argentina	ELIS A	IgG,IgM	NA	-	-	-	-	90.0 (90.0, 90.0)	100.0 (100.0, 100.0)

**47**	Core Technology Co. Ltd	Coretests COVID-19 IgM/IgG Ab Test	China	LFIA	IgG,IgM	Multiplex targets	97.6	100.0	68.9 (57, 78.7)	68.9 (57, 78.7)	67.5 (66.0, 69.0)	97.0 (97.0, 97.0)
**48**	COVIDAR	SEROKIT-ELISA COVIDAR IgG	Argentina	ELISA	IgG	NA	95.0	100.0	-	-	96.7 (96.7, 96.7)	100.0 (100.0, 100.0)
**49**	CTK Biotech, Inc.	OnSite COVID-19 IgG/IgM Rapid Test	USA	LFIA	IgG,IgM	Spike	93.4	99.4	84.6 (57.1, 100.0)	84.6 (57.1, 100.0)	90.0 (90.0, 90.0)	100.0 (100.0, 100.0)
**50**	Diagnostic Bioprobes Srl	IgG and IgM anti-SARS-CoV-2 ELISA	Italy	ELISA	IgG,IgM	Multiplex targets	98.0	98.0	-	-	-	-
**51**	DiaSorin SpA	Liaison SARS-CoV-2 S1/S2 IgG	Italy	CLIA	IgG	Spike	97.4	98.5	95.9 (87.4, 99.6)	95.9 (87.4, 99.6)	96.0 (83.0, 100.0)	98.6 (90.2, 100.0)
**52**	DiaSorin SpA	LIAISON SARS-CoV-2 S1/S2 IgM	Italy	CLIA	others	Spike	-	-	-	-	-	-
**53**	DiaSorin SpA	Liason SARS-CoV-2 IgM test	Italy	CLIA	others	Spike	-	-	91.8 (85.6, 95.5)	91.8 (85.6, 95.5)	-	-
**54**	DIAsource	anti-SARS-CoV-2 IgG ELISA	Belgium	ELISA	IgG	NA	-	-	-	-	-	-
**55**	Diazyme Laboratories Inc	Diazyme DZ-Lite SARS-CoV-2 IgG CLIA tests	USA	CLIA	IgG	Multiplex targets	97.4	98.6	100.0 (88.3, 100.0)	100.0 (88.3, 100.0)	77.8 (57.1, 98.4)	91.2 (85.0, 97.4)
**56**	Diazyme Laboratories Inc	Diazyme DZ-Lite SARS-CoV-2 IgM CLIA tests	USA	CLIA	others	Nucleocapsid	-	-	94.4 (88.4, 97.4)	94.4 (88.4, 97.4)	57.1 (57.1, 57.1)	85.0 (85.0, 85.0)
**57**	Durviz S.L.	REAL COVID19	Spain	LFIA	IgG,IgM	NA	100.0	98.0	-	-	-	-

		Rapid test cassette										
**58**	Dynamiker Biotechnology (Tianjin) Co., Ltd	2019-nCoV IgG/IgM Rapid Test	China	LFIA	IgG,IgM	Nucleocapsid	-	-	70.6 (35.3, 91.8)	70.6 (35.3, 91.8)	-	-
**59**	Eagle Bioscience	Coronavirus COVID-19 IgG ELISA Assay	USA	ELISA	IgG	Nucleocapsid	100.0	100.0	-	-	-	-
**60**	ECO Diagnostica LTDA	COVID-19 IgG/IgM ECO Test	Brazil	CLIA	IgG,IgM	Nucleocapsid	94.5	95.7	-	-	-	-
**61**	EKVITESTLAB LLC	EQUI SARS-CoV-2 IgG	Ukraine	ELISA	IgG	NA	-	-	-	-	-	-
**62**	EKVITESTLAB LLC	EQUI SARS-CoV-2 IgM	Ukraine	ELISA	others	NA	-	-	-	-	-	-
**63**	Enzo Life Sciences	SARS-CoV-2 IgG ELISA	USA	ELISA	IgG	Spike	-	-	-	-	-	-
**64**	Epitope Diagnostics, Inc.	EDI, Novel Coronavirus COVID-19 IgG ELISA Kit	USA	ELISA	IgG	Nucleocapsid	99.0	99.0	86.5 (67, 98.9)	86.5 (67, 98.9)	89.0 (89.0, 89.0)	98.0 (98.0, 98.0)
**65**	Epitope Diagnostics, Inc.	EDI, Novel Coronavirus COVID-19 IgM ELISA Kit	USA	ELISA	others	NA	-	-	65.2 (42.0, 85.4)	65.2 (42.0, 85.4)	-	-
**66**	Erba Manneim	ErbaLisa COVID-19 IgG semi quantitative kit	Germany	ELISA	IgG	Multiplex targets	-	-	-	-	-	-
**67**	ET Healthcare	Cyclic Enhanced Fluorescence Assay (CEFA)	USA	other	IgG,IgM	NA	-	-	-	-	-	-

**68**	ET Healthcare	Pylon COVID-19 IgG/IgM assay	USA	NA	IgG,IgM	Spike	-	-	-	-	89.0 (89.0, 89.0)	100.0 (100.0, 100.0)
**69**	EUROIMMUN AG	SARS-CoV-2 NCP- IgM	Germany	ELISA	others	Nucleocapsid	-	-	-	-	-	-
**70**	EUROIMMUN AG	Anti-SARS-CoV-2 ELISA (IgA)	Germany	ELISA	others	Spike	95.5	98.5	93.3 (73, 98.4)	93.3 (73, 98.4)	96.9 (85.4, 100.0)	92.5 (88.2, 99.0)
**71**	EUROIMMUN AG	Anti-SARS-CoV-2 ELISA (IgG)	Germany	ELISA	IgG	Spike	94.4	99.6	90.0 (50.0, 97.9)	90.0 (50.0, 97.9)	84.5 (55.0, 100.0)	99.3 (91.9, 100.0)
**72**	EUROIMMUN AG	SARS-CoV-2-NCP-IgG ELISA	Germany	ELISA	IgG	Nucleocapsid	94.6	99.8	72.0 (63.0, 80.0)	72.0 (63.0, 80.0)	-	-
**73**	Exsera BioLabs	Not available	USA	NA	IgG	Nucleocapsid	85.0	98.6	-	-	-	-
**74**	FBSI State scientific center for applied microbiology and biotechnology	ELISA anti-SARS-CoV reagent kit -2 IgG	Russian Federation	ELISA	IgG	Nucleocapsid	-	-	-	-	-	-
**75**	Fortress	SARS-CoV-2 ELISA	UK	ELISA	IgG,IgM,IgA	Spike	-	-	-	-	-	-
**76**	Fortress Diagnostics	Coronavirus Antibody Rapid Test	UK	LFIA	IgG,IgM	Spike	95.0	100.0	-	-	84.4 (84.4, 84.4)	98.6 (98.6, 98.6)
**77**	Genalyte (San Diego, CA)	Maverick Multi-Antigen Serology	USA	other	IgG,IgM	Multiplex targets	-	-	96.1 (92.2, 98.1)	96.1 (92.2, 98.1)	-	-
**78**	GenBody Inc.	GenBody COVID-19 IgM/IgG	Republic of Korea	LFIA	IgG,IgM	NA	74.1	100.0	58.5 (11.0, 75.4)	58.5 (11.0, 75.4)	56.7 (40.0, 60.0)	98.8 (98.8, 100.0)

**79**	Genetico	CoronaPass total antibodies test	Russian Federation	ELISA	IgG,IgM,IgA	Spike	98.7	100.0	-	-	92.0 (92.0, 92.0)	100.0 (100.0, 100.0)
**80**	Genobio Pharmaceutical (Shanghai China)	Virusee Immunochromatographic Rapid Method	China	LFIA	IgG,IgM	NA	95.3	96.8	80.0 (62.7, 90.5)	80.0 (62.7, 90.5)	-	-
**81**	Genrui Biotech	Novel Coronavirus IgG/IgM test kit	China	LFIA	IgG,IgM	NA	91.0	95.4	-	-	-	-
**82**	GenScript	cPass SARS-CoV-2 Surrogate Virus Neutralization Test ELISA Kit	USA	ELISA	IgG,IgM	Spike	-	-	100.0 (87.1, 100.0)	100.0 (87.1, 100.0)	87.9 (80.3, 95.4)	95.1 (90.8, 99.3)
**83**	Getz Pharma	IgG/IgM Test Kit (Colloidal gold)	Pakistan	NA	IgG,IgM	NA	-	-	-	-	-	-
**84**	Gold Standard Diagnostic	SARS-CoV-2 IgG ELISA Assay	USA	ELISA	IgG	Nucleocapsid	-	-	-	-	-	-
**85**	Guangzhou Wondfo Biotech Co., Ltd	Finecare SARS-CoV-2 Antibody test	China	LFIA	IgG,IgM	Spike	86.4	99.6	79.4 (61.8, 90.2)	79.4 (61.8, 90.2)	84.8 (84.8, 86.4)	99.6 (99.0, 99.9)
**86**	Guangzhou Wondfo Biotech Co.Ôºå Ltd	Wondfo SARS-CoV-2 Antibody Test	China	LFIA	IgG,IgM,IgA	NA	86.4	99.6	93.8 (93.8, 93.8)	93.8 (93.8, 93.8)	84.8 (84.8, 84.8)	99.5 (99.0, 99.9)
**87**	Hanghzhou AllTest Biotech Co., Ltd	2019-nCoV IgG/IgM Rapid Test Cassette	China	LFIA	IgG,IgM	Nucleocapsid	99.9	98.0	95.4 (26.8, 100.0)	95.4 (26.8, 100.0)	86.5 (63.6, 88.0)	100.0 (96.6, 100.0)
**88**	Hangzhou Biotest Biotech Co., Ltd	RightSign COVID-19	China	LFIA	IgG,IgM	Spike	-	-	98.0 (58.6, 100.0)	98.0 (58.6, 100.0)	84.0 (79.5, 95.0)	100.0 (100.0, 100.0)

		IgG/IgM Rapid Test Cassette										
**89**	Hangzhou Clongene Biotech Co., Ltd	Lungene 2019-nCoV IgG/IgM Rapid Test	China	LFIA	IgG,IgM	NA	97.4	98.9	97.4 (97.4, 97.4)	97.4 (97.4, 97.4)	77.1 (77.1, 77.1)	95.4 (95.4, 95.4)
**90**	Hangzhou Laihe Biotech Co., Ltd	LYHER Novel Coronavirus (2019-nCoV) IgM/IgG Antibody Combo Test Kit(Colloidal Gold)	China	LFIA	IgG,IgM	Spike	-	-	98.5 (88.7, 100.0)	98.5 (88.7, 100.0)	100.0 (100.0, 100.0)	98.8 (98.8, 98.8)
**91**	Hangzhou Realy Tech Co., Ltd	Realy-Tech 2019 nCOV/COVID-19 IgG/IgM Rapid Test	China	LFIA	IgG,IgM	Spike	97.4	96.4	92.7 (83.3, 99.4)	92.7 (83.3, 99.4)	-	-
**92**	Hangzhou Testsea Biotechnology Co., Ltd	SARS-COV-2-IgG/IgM Test Cassette	China	LFIA	IgG,IgM	Spike	92.0	100.0	-	-	-	-
**93**	Healgen	COVID-19 IgG/IgM Rapid Test Cassette	USA	LFIA	IgG,IgM	Spike	-	-	100.0 (88.7, 100.0)	100.0 (88.7, 100.0)	-	-
**94**	ID.Vet	ID Screen	France	ELISA	IgG	Nucleocapsid	-	-	-	-	92.6 (92.6, 92.6)	99.6 (99.6, 99.6)
**95**	IDEAL TASHKHIS Atieh Co	anti-SARS-CoV-2 IgG and IgM ELISA kit	Iran (Islamic Republic of)	ELISA	IgG,IgM	NA	-	-	-	-	82.0 (82.0, 82.0)	-
**96**	In3diagnostic	ERADIKIT,Ñ¢ COVID19-IgG Indirect ELISA for the detection	Italy	ELISA	IgG	NA	-	-	-	-	-	-

		of anti SARS-CoV2										
**97**	InBios International, Inc.	anti-SARS-CoV-2 IgM and IgG	USA	ELISA	IgG	Spike	-	-	100.0 (88.7, 100.0)	100.0 (88.7, 100.0)	-	-
**98**	InBios International, Inc.	anti-SARS-CoV-2 IgM	USA	ELISA	others	Spike	-	-	96.7 (83.3, 99.4)	96.7 (83.3, 99.4)	-	-
**99**	Ingenasa, Eurofins	INgezim COVID 19 DR test	Spain	ELISA	IgG,IgM,IgA	Nucleocapsid	93.0	95.0	-	-	92.0 (92.0, 92.0)	100.0 (100.0, 100.0)
**100**	Innovita Biological Technology Co. Ltd	2019-nCoV IgG Rapid Test	China	LFIA	IgG	NA	87.3	100.0	-	-	-	-
**101**	Innovita Biological Technology Co. Ltd	2019-nCoV Ab Test (Colloidal Gold) (IgM/IgG Whole Blood/Serum/Plasma Combo)	China	LFIA	IgG,IgM	NA	87.3	100.0	59.4 (44.5, 100.0)	59.4 (44.5, 100.0)	87.3 (83.3, 97.1)	97.6 (95.3, 100.0)
**102**	Institut Virion/Serion	SERION ELISA agile SARS-CoV-2	Germany	ELISA	IgG	Spike	-	-	-	-	-	-
**103**	J. Mitra & Co. Pvt., Ltd	COVID Kawach IgG Microlisa	India	ELISA	IgG	NA	-	-	-	-	-	-
**104**	Kantaro Biosciences, LLC	COVID-SeroKlir, Kantaro Semi-Quantitative SARS-CoV-2 IgG Antibody Kit	USA	ELISA	IgG	Spike	-	-	99.1 (97.0, 99.8)	99.1 (97.0, 99.8)	-	-

**105**	Karma Azma Andish Co.	COVID-19 IgM/IgG rapid test	Iran (Islamic Republic of)	LFIA	IgG,IgM	NA	-	-	-	-	71.1 (71.1, 71.1)	58.7 (58.7, 58.7)
**106**	Kovalent	KOVID Ab (COVID-19 IgG / IgM) kit	Sweden	NA	IgG,IgM	NA	95.8	97.0	-	-	-	-
**107**	Kurabo Industries Ltd	SARS-CoV-2 antibody detection kit (IgM/IgG)	Japan	NA	IgG,IgM	NA	76.4	100.0	-	-	-	-
**108**	LakePharma Inc.	SARS-CoV-2 spike protein IgG MMIA assay	USA	other	IgG	Spike	-	-	-	-	98.0 (98.0, 98.0)	100.0 (100.0, 100.0)
**109**	moLab GmbH	moscreen 2019-NCOV Corona Virus Test	Germany	LFIA	IgG,IgM	NA	-	-	-	-	98.8 (80.0, 100.0)	98.8 (97.0, 100.0)
**110**	Maccura Biotechnology Ltd	SARS CoV-2 IgM/IgG Antibody Assay Colloidal Gold Complex test	China	LFIA	IgG,IgM	Multiplex targets	-	-	-	-	-	-
**111**	Medical Systems	2019-nCoV IgM/IgG CLIA	China	CLIA	IgG,IgM	NA	-	-	-	-	25.0 (0.0, 50.0)	99.0 (98.9, 99.1)
**112**	MedLevensohn	MedTest Coronavirus 2019-nCoV IgG/IgM	Spain	LFIA	IgG,IgM	NA	85.0	99.0	-	-	-	-
**113**	MEDsan GmbH	COVID-19 IgG RDT	Germany	LFIA	IgG,IgM	Spike	-	-	90.0 (74.4, 96.5)	90.0 (74.4, 96.5)	-	-
**114**	Menarini Diagnostics	COVID-19 IgG/IgM Rapid Test Cassette	Italy	LFIA	IgG,IgM	NA	-	100.0	-	-	-	-

**115**	Meso Scale Discovery	Meso Scale Discovery multiplex assay	USA	NA	IgG	Spike	-	-	-	-	-	-
**116**	Mikrogen GmbH	recomLine SARS-CoV-2 IgG	Germany	other	IgG	Multiplex targets	-	-	-	-	-	-
**117**	Mikrogen GmbH	recomWell SARS-CoV-2 IgG ELISA	Germany	ELISA	IgG	Nucleocapsid	98.0	98.7	96.3 (90.9, 98.6)	96.3 (90.9, 98.6)	-	-
**118**	Mologic	SARS-CoV-2 ELISA Serology	UK	ELISA	IgG	NA	-	-	-	-	-	-
**119**	MP Biomedical	Rapid 2019-nCoV IgG/IgM Combo Diagnostic Test Cards	USA	LFIA	IgG,IgM	Multiplex targets	-	-	96.7 (83.3, 99.4)	96.7 (83.3, 99.4)	-	-
**120**	Multi-G B.V.	COVID-19 IgG/IgM Ab Test Cassette (Whole Blood/Serum/Plasma)	Belgium	LFIA	IgG,IgM	Nucleocapsid	-	-	-	-	92.2 (92.2, 92.2)	97.0 (97.0, 97.0)
**121**	nal von minden GmbH	NADAL COVID-19 IgG/IgM Test	Germany	LFIA	IgG,IgM	NA	94.1	99.2	-	-	100.0 (92.1, 100.0)	99.0 (97.0, 100.0)
**122**	Nanjing Vazyme Medical Technology Co., Ltd	2019-Novel Coronavirus (2019-nCoV) IgG/IgM Detection Kit	China	LFIA	IgG,IgM	Multiplex targets	91.5	97.0	96.7 (83.3, 99.4)	96.7 (83.3, 99.4)	-	-
**123**	NG Biotech Laboratoires	NG-Test finger-prick test	France	LFIA	IgG,IgM	NA	100.0	95.3	91.8 (84.5, 95.9)	91.8 (84.5, 95.9)	82.5 (82.5, 82.5)	98.0 (98.0, 98.0)
**124**	NovaTec Immundiagnostics GmbH	NovaLisa SARS-CoV-2 IgG	Germany	ELISA	IgG	NA	100.0	100.0	77.6 (55.0, 96.7)	77.6 (55.0, 96.7)	94.9 (94.9, 94.9)	96.2 (96.2, 96.2)

**125**	NZK Biotech Co., Wuhan, China	dual-target immuno-fluorescence assay	China	LFIA	IgG,IgM	Multiplex targets	0.0	0.0	-	-	-	-
**126**	Omega diagnostics	COVID-19 IgG ELISA	UK	ELISA	IgG	Multiplex targets	0.0	0.0	-	-	88.1 (88.1, 88.1)	93.2 (93.2, 93.2)
**127**	Ortho Clinical Diagnostics Inc.	VITROS Immunodiagnostic Products Anti-SARS-CoV-2 IgG	USA	CLIA	IgG	Spike	90.0	100.0	94.5 (88.7, 100.0)	94.5 (88.7, 100.0)	93.6 (90.4, 100.0)	100.0 (99.6, 100.0)
**128**	Ortho Clinical Diagnostics Inc.	VITROS Immunodiagnostic Products Anti-SARS-CoV-2 Total	USA	CLIA	IgG,IgM,IgA	Spike	90.0	100.0	100.0 (92.7, 100.0)	100.0 (92.7, 100.0)	93.2 (85, 100.0)	99.4 (99.0, 100.0)
**129**	Palex	Diapro	Spain	ELISA	IgG	Multiplex targets	98.0	90.0	-	-	88.9 (88.9, 88.9)	91.8 (91.8, 91.8)
**130**	PCL Inc	PCL COVID19 IgG/IgM Rapid Gold	Republic of Korea	NA	IgG,IgM	NA	-	-	83.0 (83.3, 99.4)	83.0 (83.3, 99.4)	-	-
**131**	PCL Inc	PCL COVID-19 Total Ab EIA test	Republic of Korea	ELISA	IgG,IgM,IgA	Multiplex targets	98.2	100.0	-	-	-	-
**132**	PerkinElmer	GSP1/DELFIA 1 anti SARS-CoV2 kit	Italy	LFIA	IgG	Spike	96.0	100.0	-	-	-	-
**133**	Phamatech	COVID19 RAPID TEST	USA	NA	IgG,IgM	NA	-	-	86.7 (70.3, 94.7)	86.7 (70.3, 94.7)	-	-
**134**	PharmACT	BELTEST-IT COV-2 Rapid Test	Germany	FIA	IgG,IgM	NA	-	-	-	-	-	-
**135**	Pishtaz Diagnostics Iran	ELISA kit Pishtaz Teb Diagnostics	Iran (Islamic	ELISA	IgG	Nucleocapsid	94.1	98.3	-	-	76.7 (59.0, 100.0)	98.2 (97.3, 100.0)

			Republic of)									
**136**	Pishtaz Diagnostics Iran	ELISA kit Pishtaz Teb Diagnostics	Iran (Islamic Republic of)	ELISA	others	Nucleocapsid	-	-	-	-	-	-
**137**	Polycheck	anti-SARS-CoV-2 IgG immunoblot	Germany	other	IgG	Multiplex targets	-	-	-	-	-	-
**138**	Premier Biotech	COVID-19 IgG/IgM Rapid Test Cassette	USA	LFIA	IgG,IgM	NA	-	-	-	-	84.1 (82.7, 85.6)	99.5 (99.5, 99.5)
**139**	PRIMA Lab S.A.	PRIMA COVID-19 IgG/IgM Rapid Test	Switzerland	NA	IgG,IgM	Multiplex targets	100.0	98.0	-	-	100.0 (100.0, 100.0)	97.8 (96.0, 99.6)
**140**	Prognosis Biotech	Rapid Test 2019-nCoV Total Ig	Greece	LFIA	IgG,IgM,IgA	Spike	98.8	100.0	-	-	-	-
**141**	Qingdao Hightop Biotech Co., Ltd	Hightop COVID-19 IgM/IgG Ab Rapid Test Kit	China	LFIA	IgG,IgM	NA	-	-	81.8 (59.5, 78.2)	81.8 (59.5, 78.2)	95.0 (95.0, 95.0)	100.0 (100.0, 100.0)
**142**	Quotient	MosaiQ COVID-19 Antibody Microarray	Switzerland	other	IgG,IgM	Spike	-	-	93.0 (85.6, 96.8)	93.0 (85.6, 96.8)	-	-
**143**	RayBiotech	Novel Coronavirus IgG antibody test kit (colloidal gold method)	USA	ELISA	IgG	NA	90.4	98.3	70.0 (52.1, 83.3)	70.0 (52.1, 83.3)	76.0 (76.0, 76.0)	95.0 (95.0, 95.0)
**144**	Roche Diagnostics	Elecsys®Anti-SARS-CoV-2 (N)	Switzerland	CLIA	IgG,IgM,IgA	Nucleocapsid	99.5	99.8	100.0 (88.3, 100.0)	100.0 (88.3, 100.0)	96.8 (83.9, 100.0)	99.8 (83.9, 100.0)

**145**	Roche Diagnostics	Elecsys®Anti-SARS-CoV-2 (S)	Switzerland	CLIA	IgG,IgM,IgA	Spike	98.8	99.6	95.6 (90.3, 98.3)	95.6 (90.3, 98.3)	97.9 (96.6, 99.5)	99.9 (99.8, 99.9)
**146**	Salofa Oy manufacturer, Finland	Sienna-Clarity COVIBLOCK COVID-19 IgG/IgM Rapid Test Cassette	Finland	LFIA	IgG,IgM	NA	-	-	93.3 (78.7, 98.2)	93.3 (78.7, 98.2)	-	-
**147**	Sci LifeLab / KTH	Not available	Sweden	ELISA	NA	Multiplex targets	99.4	98.9	-	-	98.9 (98.9, 98.9)	99.4 (99.4, 99.4)
**148**	Screen Italia S.R.L	Screen Test COVID-19 2019-nCOV IgG/IgM	Italy	LFIA	IgG,IgM	NA	-	-	-	-	90.5 (72.0, 99.0)	85.5 (83.0, 95.0)
**149**	SD Biosensor	Standard Q COVID-19 IgM/IgG Combo	Republic of Korea	FIA	IgG,IgM	NA	94.5	95.7	-	-	-	-
**150**	SD Biosensor	Standard Q COVID-19 IgM/IgG Duo rapid immunochromatography test kit	Republic of Korea	LFIA	IgG,IgM	Nucleocapsid	94.3	96.6	76.7 (57.4, 94.6)	76.7 (57.4, 94.6)	-	-
**151**	SeraCare Life Sciences	HRP-conjugated anti human IgM/IgA/IgG	USA	ELISA	IgG,IgM,IgA	Spike	-	-	-	-	-	-
**152**	Serimmune	Serum Epitope Repertoire Analysis (SERA)	USA	other	IgG,IgM,IgA	Multiplex targets	91.0	99.3	-	-	-	-
**153**	Shanghai Kehua Bio-engineering Co., Ltd	Diagnostic kit for SARS-CoV-2 IgM/IgG Antibody	China	LFIA	IgG,IgM	NA	98.8	98.0	75.3 (65.4, 83.1)	75.3 (65.4, 83.1)	95.1 (95.1, 95.1)	99.3 (99.3, 99.3)

		(Colloidal Gold)										
**154**	Shenzhen Mindray Bio-Medical Electronics Co.	Mindray CL-900i anti-SARS-CoV-2 IgG	China	CLIA	IgG	Multiplex targets	-	-	-	-	-	-
**155**	Shenzhen Sciarray Biotech Ltd	ELISA test	China	ELISA	IgG,IgM,IgA	Nucleocapsid	-	-	-	-	-	-
**156**	Shenzhen Yhlo Biotech Co. Ltd	iFlash-SARS-CoV-2 IgM/IgG	China	CLIA	IgG,IgM	Multiplex targets	94.3	97.0	-	-	95.5 (88.2, 100.0)	99.3 (92.9, 100.0)
**157**	Shin Jin Medics Inc	DIAKEY COVID-19 IgM/IgG Rapid Test Kit	Republic of Korea	LFIA	IgG,IgM	Multiplex targets	-	-	-	-	100.0 (100.0, 100.0)	92.0 (92.0, 92.0)
**158**	Siemens	ADVIA Centaur Immunoassay System	Germany	CLIA	IgG,IgM,IgA	Spike	100.0	99.8	100.0 (91.6, 100.0)	100.0 (91.6, 100.0)	92.3 (86.0, 98.1)	99.5 (99.0, 99.9)
**159**	Siemens	Atellica IM SARS-CoV-2 Total (COV2T)	Germany	CLIA	IgG,IgM,IgA	Spike	100.0	99.9	96.3 (86.1, 100.0)	96.3 (86.1, 100.0)	95.7 (95.0, 96.4)	99.9 (99.9, 99.9)
**160**	Snibe Co., Ltd (Shenzhen New Industries Biomedical Engineering Co., Ltd)	MAGLUMI 2019-nCoV IgM/IgG	China	CLIA	IgG,IgM	Multiplex targets	91.2	97.3	100.0 (86.2, 100.0)	100.0 (86.2, 100.0)	95.6 (73.0, 100.0)	98.8 (96, 100.0)
**161**	Sugentech, Inc.	SGTi-flex COVID-19 IgM/IgG (manual)	Republic of Korea	LFIA	IgG,IgM	NA	-	-	96.7 (83.3, 99.4)	96.7 (83.3, 99.4)	-	-

**162**	Superbio Biomedical Company	COVID-19 Rapid Test Kit IgG + IgM	China	CLIA	IgG,IgM	NA	100.0	83.8	100.0 (88.7, 100.0)	100.0 (88.7, 100.0)	-	-
**163**	SureScreen Diagnostics	COVID-19 antibody rapid test	UK	LFIA	IgG,IgM	Spike	-	-	-	-	84.4 (74.7, 94.0)	95.7 (95.1, 96.3)
**164**	Synlab	Quantitative IgG antibody test for coronavirus	Germany	CLIA	IgG	Spike	-	-	-	-	-	-
**165**	Syntron Bioresearch Inc.	Rapid lgM-lgG Combined Antibody Test for Coronavirus	USA	LFIA	IgG,IgM	Multiplex targets	-	-	-	-	-	-
**166**	Sysmex	Nucleocapsid HISCL,5000/HISCL-800 Automated Immunoassay Systems	Japan	CLIA	IgG	Nucleocapsid	-	-	-	-	-	-
**167**	Sysmex	Spike HISCL,5000/HISCL-800 Automated Immunoassay Systems	Japan	CLIA	IgG	Spike	-	-	-	-	-	-
**168**	Tecan	SARS-CoV-2 ELISA IgM and IgG	Switzerland	ELISA	IgG,IgM	NA	-	-	-	-	-	-
**169**	Technoclone	5600100 TECHNOZYM anti SARS-CoV-2 RBD IgG ELISA 96T	Austria	ELISA	IgG	Multiplex targets	-	-	-	-	-	-
**170**	Technogenetics srl	TechnoGenetics COVID-19 IgG	Italy	CLIA	IgG	Multiplex targets	100.0	98.6	-	-	-	-

**171**	Technogenetics srl	TechnoGenetics COVID-19 IgM	Italy	CLIA	others	Multiplex targets	100.0	98.6	-	-	-	-
**172**	Tecno Bios srl	SARS-CoV-2 IgG/IgM ELISA Kit IMMUNO-COVID19	Italy	ELISA	IgG,IgM	Nucleocapsid	-	-	-	-	-	-
**173**	TestLine Clinical Diagnostics	Microblot-Array COVID-19 IgA	Czechia	other	others	Multiplex targets	-	-	-	-	-	-
**174**	TestLine Clinical Diagnostics	Microblot-Array COVID-19 IgG	Czechia	other	IgG	Multiplex targets	-	-	-	-	-	-
**175**	The Binding Site	Human Anti-IgG/A/M SARS-CoV-2 ELISA	UK	ELISA	IgG,IgM ,IgA	Spike	-	-	-	-	98.6 (98.6, 98.6)	98.3 (98.3, 98.3)
**176**	TODA Pharma	TODA Coronadiag +	Japan	LFIA	IgG,IgM	NA	-	-	-	-	-	-
**177**	TONYAR Biotech Inc., Taiwan	ASK COVID-19 IgG/IgM Rapid Test	China	LFIA	IgG,IgM	NA	-	-	-	-	-	-
**178**	Trinity Biotech	Captia Anti-SARS-CoV-2 (IgG) ELISA	Ireland	ELISA	IgG	Spike	95.9	100.0	-	-	-	-
**179**	Vector BEST	SARS- CoV-2-IgG-EIA-BEST and SARS-CoV-2- IgM-EIA-BEST	Russian Federation	ELISA	IgG,IgM	NA	-	-	-	-	-	-
**180**	Vircell S.L.	COVID-19 ELISA IgG	Spain	ELISA	IgG	Multiplex targets	88.0	99.0	95.1 (90.8, 98.9)	95.1 (90.8, 98.9)	97.0 (92.5, 100.0)	94.5 (94.0, 99.2)

**181**	Vircell S.L.	COVID-19 VIRCLIA IgG MONOTEST	Spain	CLIA	IgG,IgM ,IgA	Multiplex targets	-	-	-	-	-	-
**182**	Vircell S.L.	COVID-19 ELISA IgM/IgA	Spain	ELISA	others	Multiplex targets	85.0	98.5	96.7 (90.8, 98.9)	96.7 (90.8, 98.9)	94.8 (87.5, 100.0)	95.0 (82.0, 99.2)
**183**	VivaChek Biotech (Hangzhou) Co., Ltd	VivaDiag COVID-19 IgM/IgG Rapid Test	China	LFIA	IgG,IgM	NA	-	-	88.5 (53.3, 100.0)	88.5 (53.3, 100.0)	37.6 (34.9, 83.3)	97.6 (95.7, 99.0)
**184**	Wuhan UNscience Biotechnology Co., Ltd	UNSCIENCE COVID-19 IgG/IgM antibody Rapid Test Kit	China	LFIA	IgG,IgM	NA	98.5	88.2	-	-	66.0 (66.0, 66.0)	94.0 (94.0, 94.0)
**185**	Xiamen Biotime Biotechnology Co., Ltd	SARS-CoV-2 IgG/IgM Rapid Qualitative Test Kit	China	LFIA	IgG,IgM	NA	80.4	99.4	97.0 (88.7, 100.0)	97.0 (88.7, 100.0)	-	-
**186**	ZenTech, Angleur, Belgium	QuickZen COVID-19 IgM/IgG kit	Belgium	LFIA	IgG,IgM	Spike	-	-	55.1 (44.0, 65.1)	55.1 (44.0, 65.1)	59.0 (49.2, 68.8)	100.0 (100.0, 100.0)
**187**	ZetaGene Ltd.	ZetaGene COVID-19 Antibody Test IgM/IgG	Sweden	LFIA	IgG,IgM	NA	-	-	-	-	95.0 (95.0, 95.0)	97.0 (97.0, 97.0)
**188**	Zeus	ELISA SARS-CoV-2 IgG Test System	USA	ELISA	IgG	Nucleocapsid	-	-	93.3 (78.7, 98.2)	93.3 (78.7, 98.2)	-	-
**189**	Zhejiang Orient Gene Biotech	COVID-19 IgG/IgM Rapid Test	China	LFIA	IgG,IgM	Spike	87.9	99.0	90.5 (60.2, 97.3)	90.5 (60.2, 97.3)	93.6 (69.0, 100.0)	99.2 (88.0, 100.0)
**190**	Zhuhai Livzon Diagnostics Inc	2019-nCoV IgG/IgM Antibody Detection Kit	China	LFIA	IgG,IgM	Nucleocapsid	-	-	67.3 (57.2, 98.2)	67.3 (57.2, 98.2)	82.6 (59.1, 95.8)	99.5 (77.3, 100.0)

**191**	Zybio Inc.	SARS-CoV-2 IgM/IgG Antibody Assay Kit	China	LFIA	IgG,IgM	NA	-	-	-	-	-	-

References

[1] US Food & Drug Administration. (n.d.). *openFDA*. openFDA. Retrieved March 22, 2022, from https://open.fda.gov/apis/device/covid19serology/

[2] FIND Diagnostics. (2020, April 27). *Comparative evaluation of lateral flow assay (LFA) and ELISA tests that detect human antibodies specific to SARS-CoV-2 to suppor*. finddx.org. Retrieved March 22, 2022, from https://www.finddx.org/wp-content/uploads/2020/04/20200427-COVID-19-IA-Evaluation-Synopsis.pdf

[3] FIND Diagnostics. (n.d.). *Overview of FIND methodology for antibody evaluations Introduction: Overview of the Studies:*. finddx.org. Retrieved March 22, 2022, from https://www.finddx.org/wp-content/uploads/2021/04/Overview-of-FIND-Methodology-for-Antibody-Evaluations-2021April29.pdf

[4] van den Beld, M. (2021). Increasing the Efficiency of a National Laboratory Response to COVID-19: a Nationwide Multicenter Evaluation of 47 Commercial SARS-CoV-2 Immunoassays by 41 Laboratories. *Journal of Clinical Microbiology*, *59*(9), e00767-21.

[5] Department of Health, Australian Government. (n.d.). *Post-market validation of serological point-of-care tests for COVID-19*. Australian Government Department of Health. Retrieved March 22, 2022, from https://www.health.gov.au/resources/collections/post-market-validation-of-serological-point-of-care-tests-for-covid-19

[6] Department of Health, Australian Government. (2021, March 11). *Post-market evaluation of serology-based point of care tests*. Therapeutic Goods Administration (TGA). Retrieved March 22, 2022, from https://www.tga.gov.au/post-market-evaluation-serology-based-point-care-tests

[7] An NRL and WHO Initiative. (n.d.). *SARS-CoV-2 Antibody Detection Test Kit Performance Evaluation Protocol*. nrlquality. Retrieved 3 22, 2022, from https://www.nrlquality.org.au/_files/ugd/188553_d050808aa3374506968498af59ed5ebc.pdf
